# Supplementary material for: A Syndecan‐Based Genetic Approach to Coat the Surface of Small Extracellular Vesicles With Nanobodies
Source: J Extracell Biol. 2026 May 5;5(5):e70133. doi: 10.1002/jex2.70133 (PMC13145340; doi:10.1002/jex2.70133)
Supplement: Supplementary file 1 — Supplementary Material: jex270133‐sup‐0001‐SuppMat.docx [file JEX2-5-e70133-s003.docx]

**Supplementary figures**

**
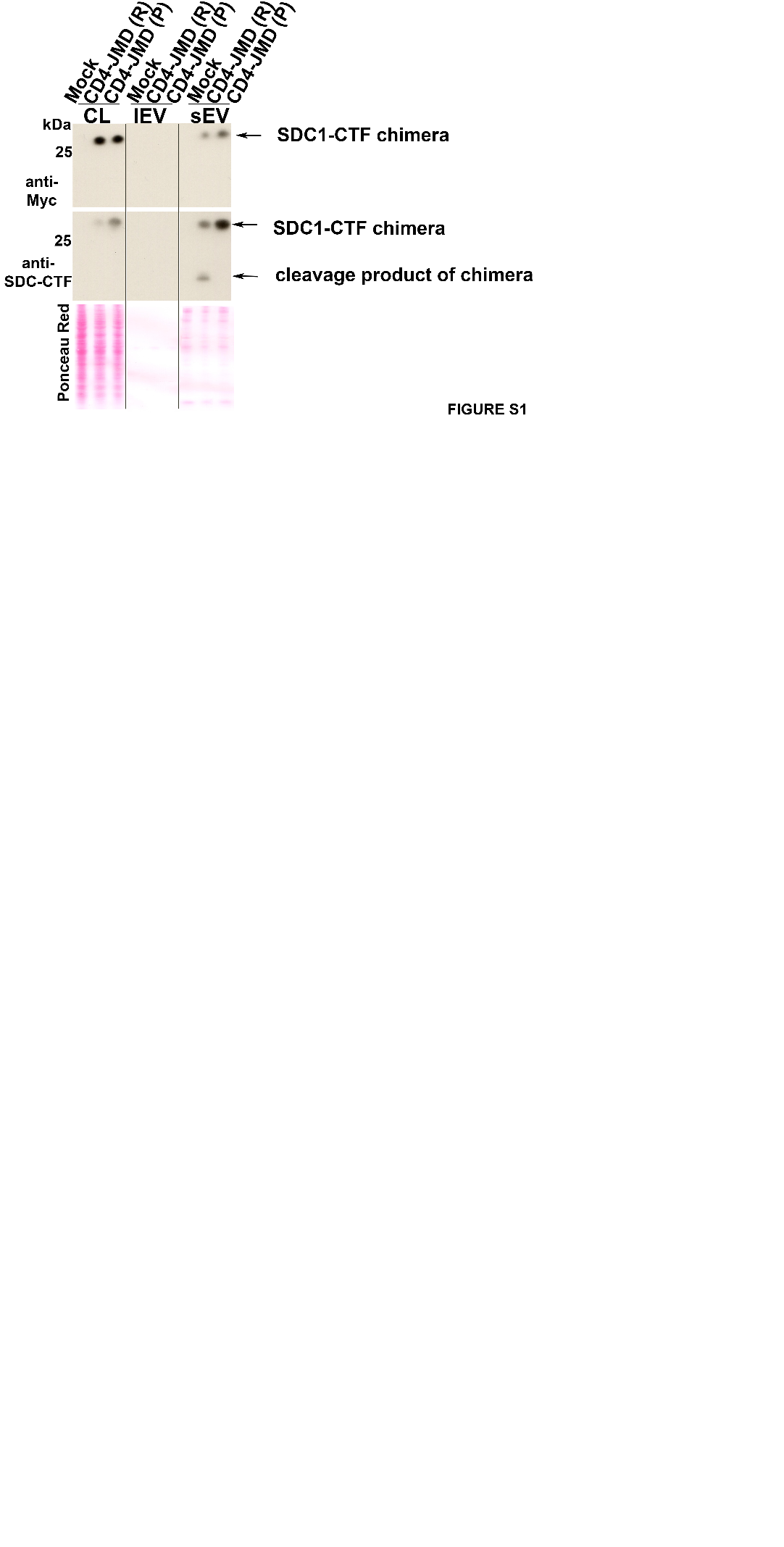

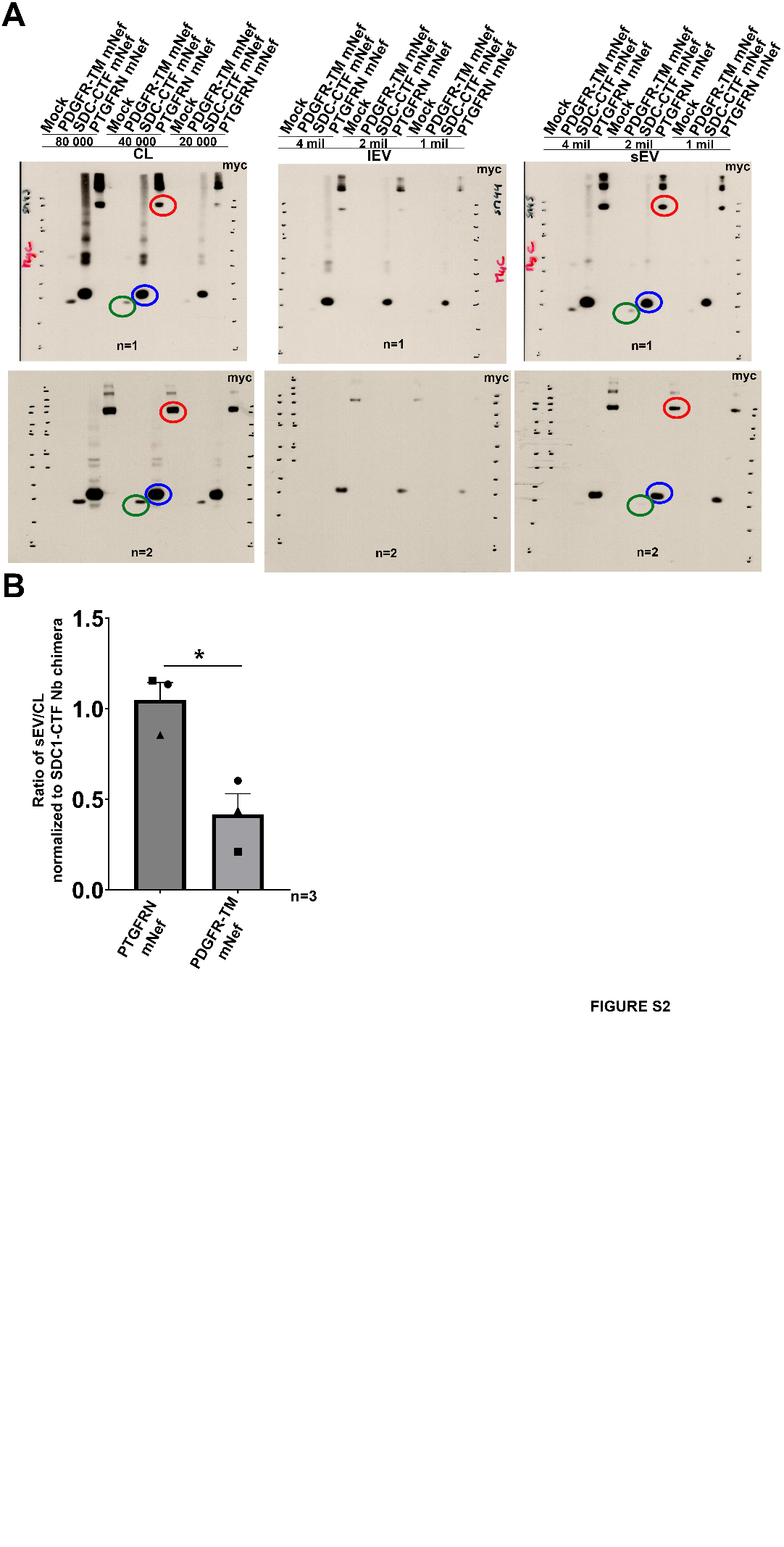

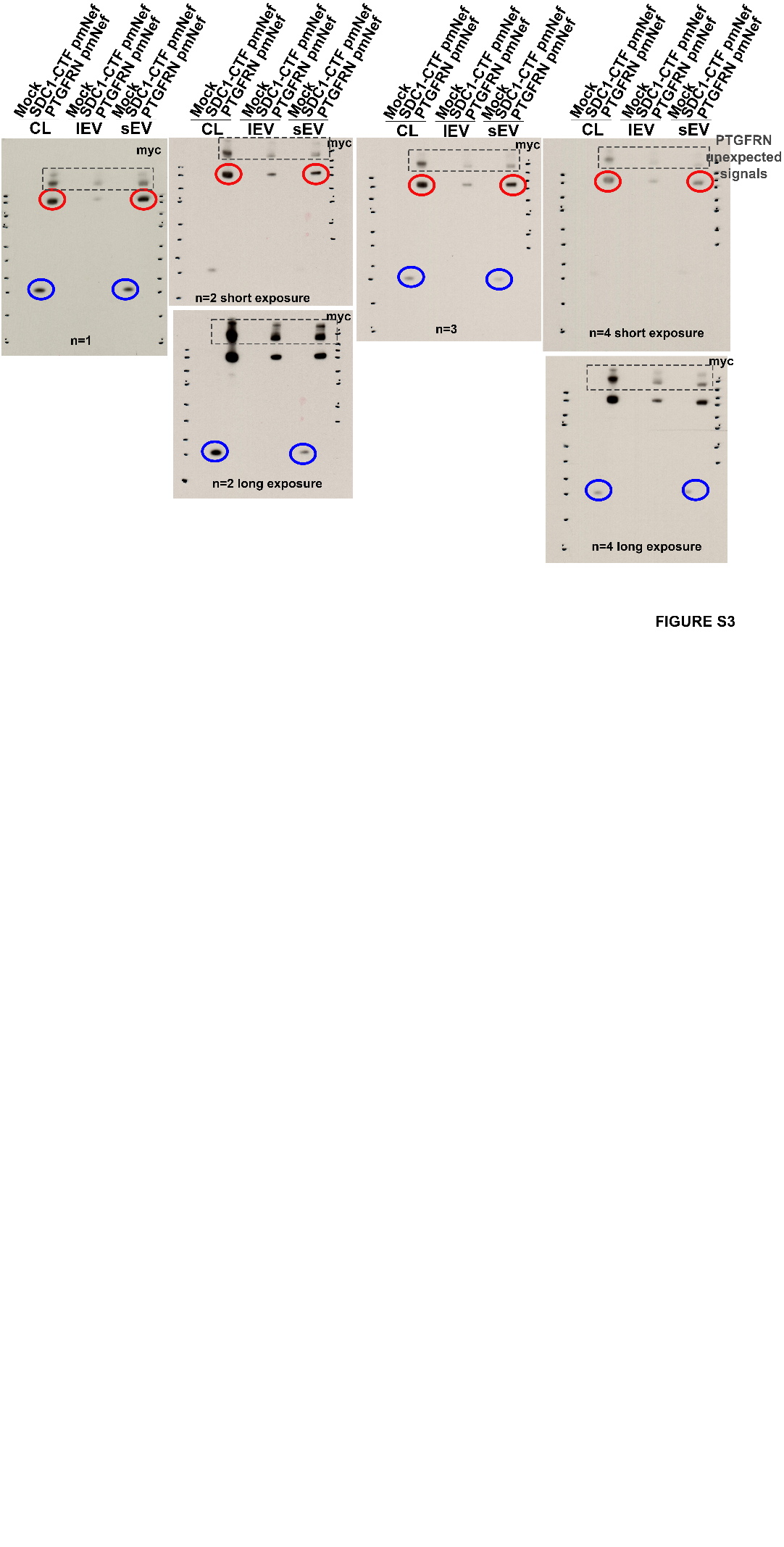

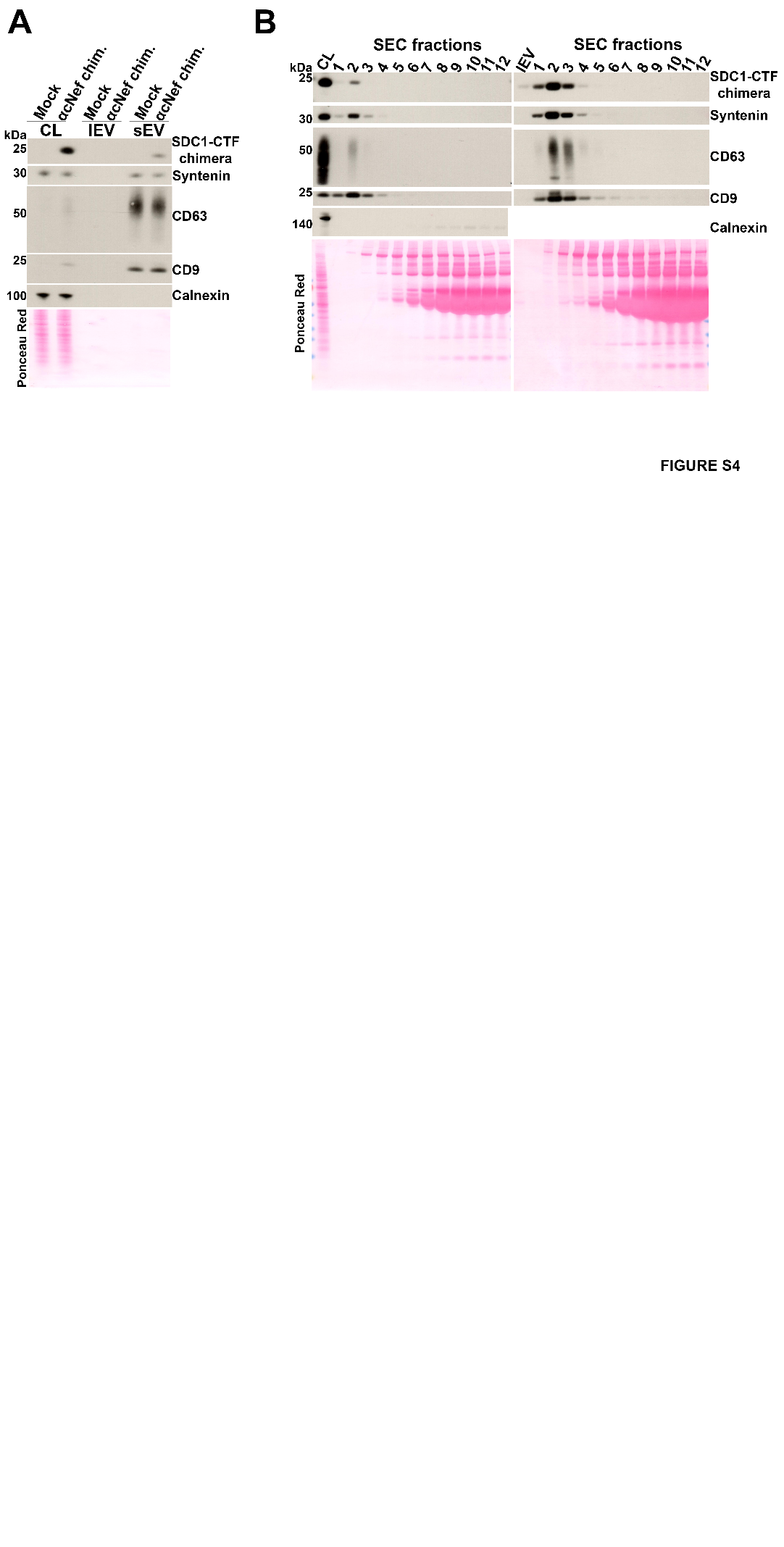

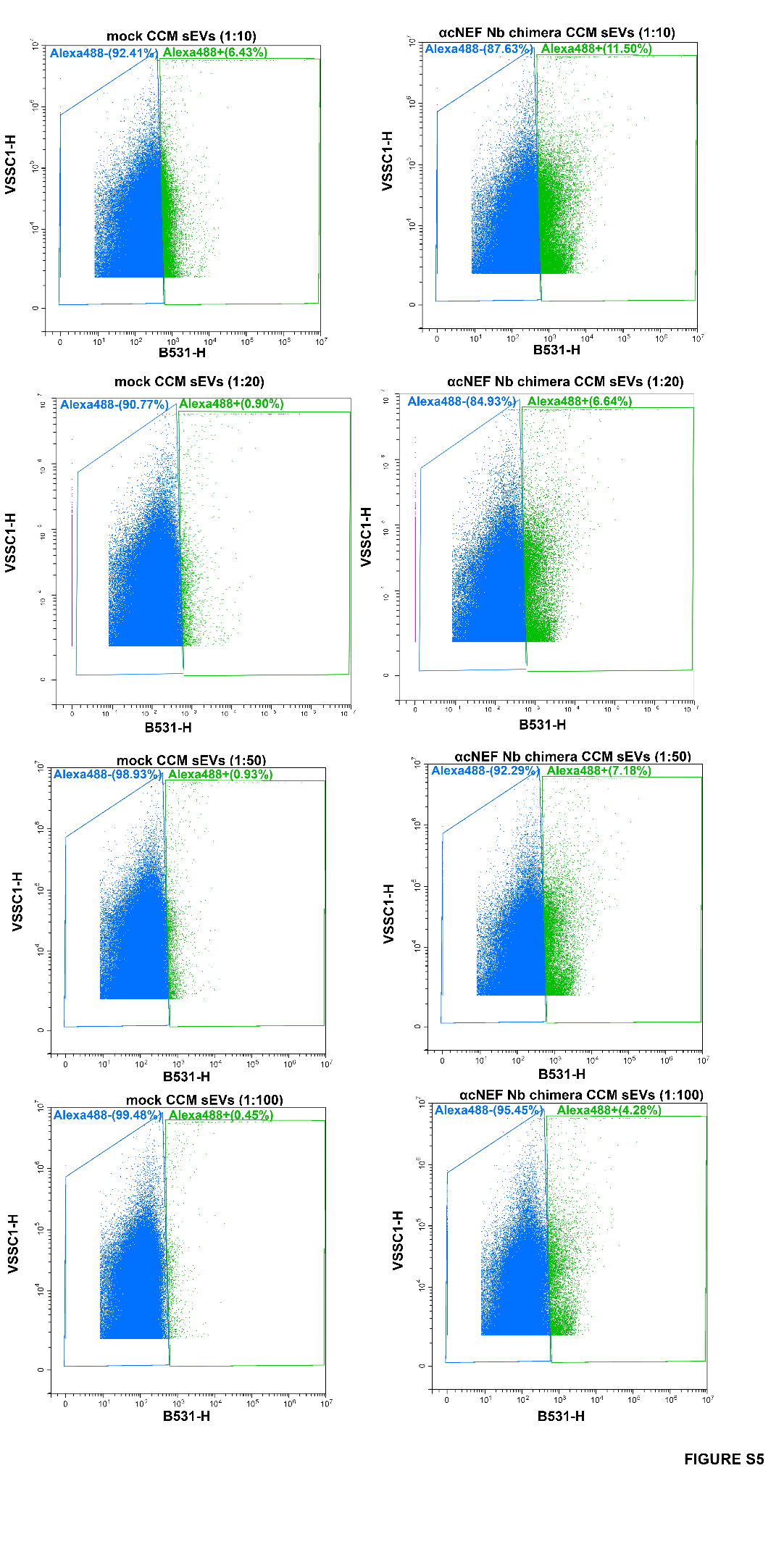

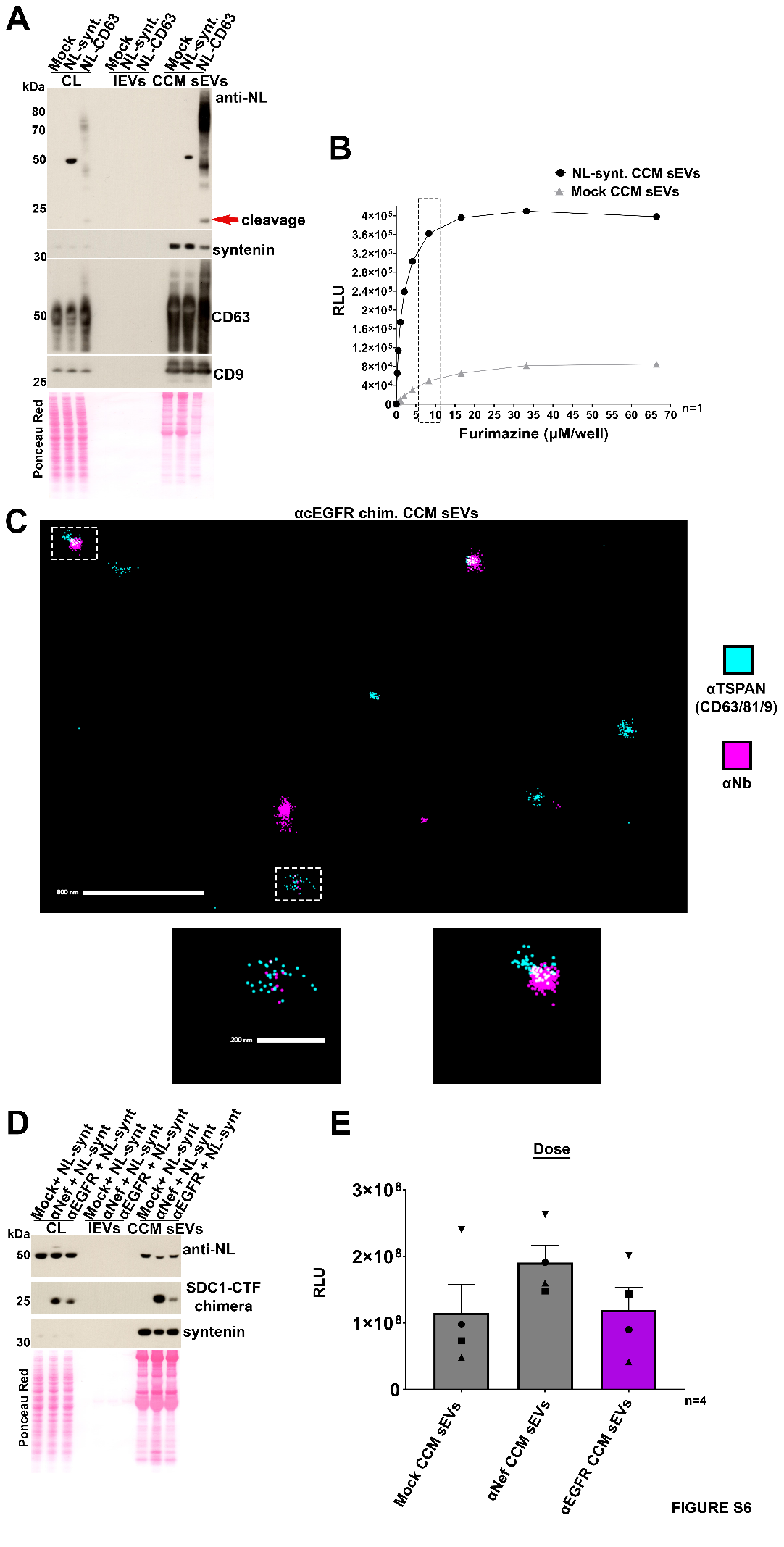

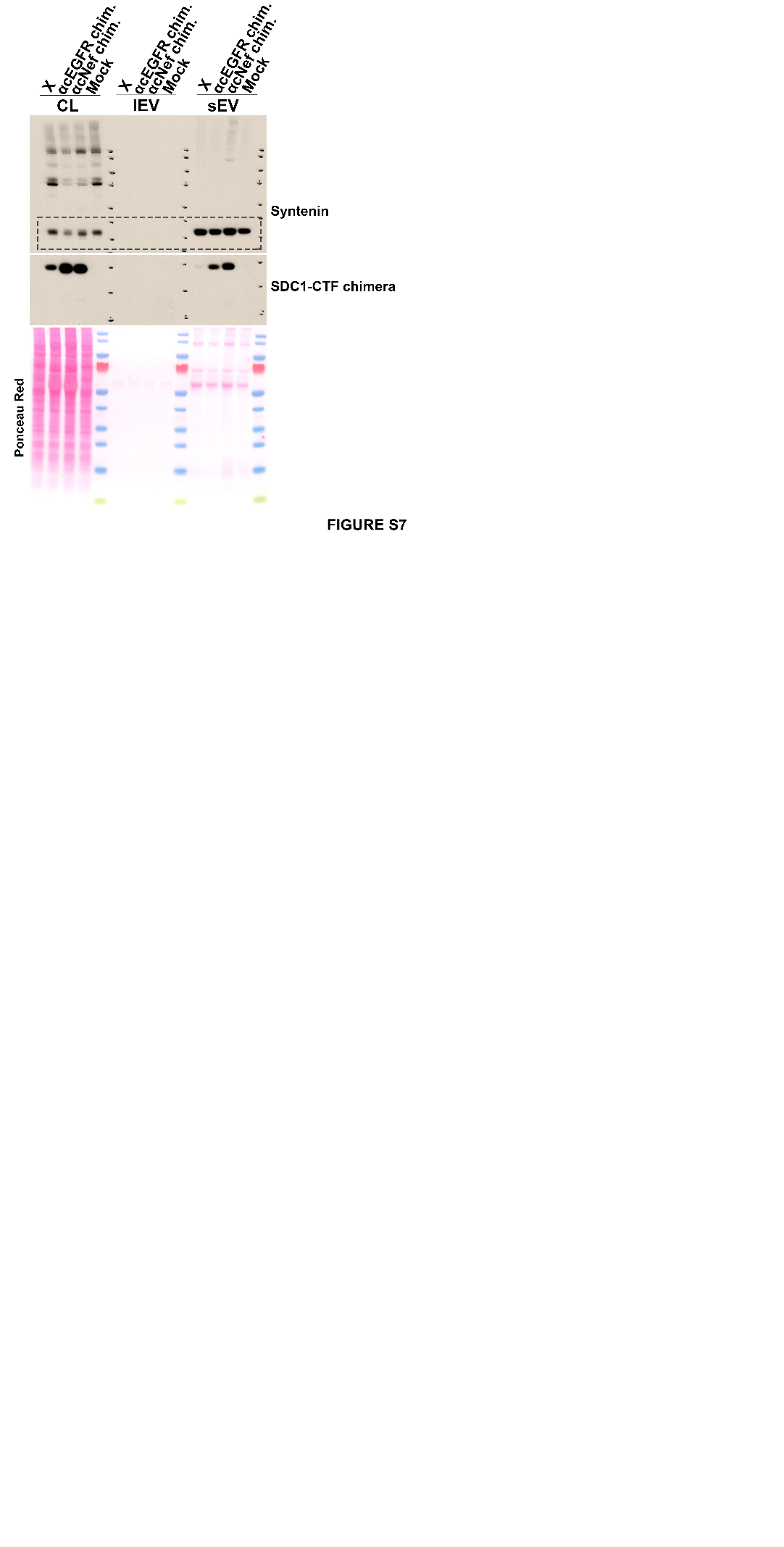
**

**Legends Supplementary figures**

**Figure S1. Impact of the CD4 juxta-membrane sequence on the cleavage of SDC1-CTF chimera.** Illustrative Western blot of cell lysates (CL) and EVs (fractionated by dUC) from HEK293 cells transiently expressing myc-tagged Nef Nb chimera containing a CD4 juxta-membrane domain with an arginine (CD4-JMD (R)) or a proline (CD4-JMD (P)). Large EV (lEV) and small EV (sEV) fractions correspond to the 10K and 100K pellets, respectively. Blots were probed with anti-myc antibodies (upper panel), an epitope present at the N-terminus of the chimera, or anti-SDC1 antibodies (middle panel) recognizing the C-terminus of the chimera. CL correspond to 20.000 cells. Secretomes were collected from the conditioned media of 3.6 x 10^6^ cells. Red Ponceau (lower panel) was used as loading control. Images are from the same blot. Note the presence of a cleavage product in the sEV fraction of CD4-JMD (R) transfectants. Related to **Fig. 1A.**

**Figure S2: PDGFR-TM chimera is not efficient at sorting Nanobodies to extracellular vesicles. (A)** Uncropped Western blots of HEK293 cell lysates (CL), and large and small EV (lEV, sEV) from cells transiently transfected with various constructs as indicated. Mock refers to cells transfected with an empty vector. For CL, numbers refer to cell amounts. For lEV and sEV fractions, numbers correspond to the amount of producing cells. Anti-myc antibodies were used to compare the distribution of SDC1-CTF, PTGFRN, and PDGFR-TM myc-tagged Nef Nbs, as indicated. Note the unexpected signals detected in SDC1-CTF and PTGFRN transfectants. Circles indicate areas used for quantification of sorting efficiencies. Blue for SDC1-CTF, red for PTGFRN and green for PDGFR-TM chimeras. **(B)** Histogram illustrating that PDGFR-TM chimera is less efficient at sorting Nbs to sEVs than SDC1-CTF chimera. Anti-myc signals obtained in Western blot for sEV fractions and CL, were normalized to SDC1-CTF chimera signals in their respective fractions and expressed as a mean ratio sEV/CL from 3 independent experiments. Bars represent mean values + SEM. Student’s t-test was applied to assess statistical significance. Related to **Fig. 1B-C.**

**Figure S3. Unexpected signals remain present in cells stably overexpressing the PTGFRN chimera.** Uncropped Western blots of HEK293 cell lysates (CL), and large and small EVs (lEV, sEV), from cells stably overexpressing SDC1-CTF and PTGFRN chimeras. Mock refers to cells transfected with an empty vector. CL correspond to 20.000 cells. EVs were collected from the conditioned media of 3.6 x 10^6^ cells. Circles indicate areas used for the quantification of sorting efficiencies of SDC1-CTF (blue) and PTGFRN (red) chimeras. Related to **Fig. 2A.**

**Figure S4. dUC characterization of cNef and additional SEC blots. (A)** Illustrative Western blot of EVs fractionated by dUC (lEVs and sEVs pellets, as indicated), along with the corresponding cell lysates (CL) from the HEK293 clone stably overexpressing the SDC1-CTF Nef Nb chimera. Mock refers to HEK293 cells stably transfected with an empty vector. Various EV marker proteins used as positive controls and calnexin used as negative control, as indicated on the right. Ponceau red was used as loading and transfer control. Note that the chimera is detected in CL and sEVs but is absent from lEVs. **(B)** Western blots showing two extra biological repeats of sEV fractionation by SEC experiments as in **Fig. 3D**. Related to **Fig. 3** and **Fig. 5.**

**Figure S5. Nano flow analysis data.** Dot plots of CCM sEVs from mock-transfected (left) or anti-Nef CCM sEVs (right). CCM sEVs were stained with Alexa488-conjugated anti-Nb antibody, using different dilutions. The gating strategy was used to separate the bulk of dense signal (blue) of the Mock CCM sEVs treated with 1:20 antibody dilution (concentration used throughout this study for single-EV detection) and transposed to all the other samples. Percentages of Nb negative (blue) and positive (green) particles are indicated. Related to **Fig. 3.**

**Figure S6. Characterization of Nanoluciferase-syntenin loaded CCM sEVs by Western blot and luminescence. Single-vesicle characterization of anti-EGFR CCM sEVs. (A)** Representative Western blots of the cell lysates (CL) and secretomes (lEV and CCM sEVs) of HEK293 cells transiently overexpressing Nanoluciferase-syntenin (NL-synt) or Nanoluciferase-CD63 (NL-CD63), as indicated. Mock refers to HEK293 cells transiently transfected with an empty vector. Blots were probed with anti-NL (upper panel) or sEV marker antibodies, as indicated on the right. Ponceau red was used as loading and transfer control. Note that both NL-synt and NL-CD63 are efficiently sorted to CCM sEVs. Also note the low-molecular-weight signal (red arrow) in the NL-CD63 CCM sEVs suggesting cleavage of the NL part from the NL-CD63. CL correspond to 20.000 cells. Secretomes were collected from the conditioned media of 4.8 x 10^6^ cells. **(B)** Dose-response curve illustrating the luminescence signals (relative light units, RLU, Y-axis) obtained with NL-synt loaded (black) or mock CCM sEVs (grey) at increasing concentrations (µM/well, X-axis) of the Nanoluciferase substrate, Furimazine. From there, we decided to set the Furimazine concentration at 8.3 µM (indicated in dashed frame). **(C)** Representative micrographs of anti-EGFR CCM sEVs characterized by ONI® microscopy. Anti-tetraspanin (CD9, CD63, CD81) signals are in cyan while anti-Nb signals are in magenta. Scale bars correspond to 800 nm for field view and 200 nm for inserts. **(D)** Illustrative Western blot of CCM sEVs prepared from the different SDC1-CTF chimera clones also overexpressing NL-syntenin, as indicated on the top. Blots were probed with anti-NL antibodies, anti-SDC1 antibodies recognizing the intracellular domain of SDC1, and anti-syntenin antibodies recognizing the endogenous syntenin. Ponceau red was used as loading and transfer control. CL corresponds to 20.000 cells. The secretome was prepared from conditioned media of 2.4 x 10^6^ cells. **(E)** Bar graph showing the luminescence signals (RLU, Y-axis) from NL-syntenin loaded CCM sEVs obtained from cells stably expressing the different SDC1-CTF chimeras (as indicated on the X-axis). Individual points represent individual doses administered to Panc-1 cells in 4 independent biological repeats. Bars show mean values + SEM. Related to **Fig. 5.**

**Figure S7. Uncropped blot of cNef and cEGFR cells.** Uncropped western blot of the cell lysates (CL), large EV (lEV) and small EV (sEV) fractions (obtained by dUC) of cNef and cEGFR HEK293 cells. Dashed area shows cropped part of the anti-syntenin blot used in **Fig. 5.**

We would like to thank the reviewers and editors for their thorough assessment of our manuscript and for recognizing the technical rigor and clarity of our study. We carefully considered all points raised and have substantially revised the text to better position our work, clarify novelty, and acknowledge limitations. Below, we provide a detailed response to each comment.

_________________________________________________________________________________________

**Reviewer 1**

**Comment:** The manuscript does not advance the field in a major way, nor offer particular novel insights.
**Response:** We respectfully disagree that the manuscript lacks novelty. While genetic engineering for nanobody display on EVs has been explored previously, our approach introduces several distinct contributions: (i) use of the SDC1-CTF fusion protein as a compact and efficient transmembrane anchor; (ii) incorporation of a protease-resistant linker that ensures stable nanobody display; and (iii) systematic testing with single-vesicle characterization methods. We have revised the manuscript to more clearly situate our work within the existing literature and highlight these specific advances.

**Comment:** Uptake assays lack depth and follow-up, and Nanoluc signal may reflect binding rather than uptake.
**Response:** We acknowledge this limitation and now state it explicitly. Importantly, we define uptake as both cell-surface binding and internalization, since EVs may signal through either mechanism. While we agree that future work should include co-cultures and EGFR-negative cells, these experiments are beyond the scope of the current study, which primarily aimed to establish the feasibility of SDC1-CTF nanobody decoration and benchmark it with existing scaffolds.

**Comment:** Nanoluc uptake normalization should be based on Nanoluc activity rather than particle count.
**Response:** We thank the reviewer for this suggestion. In our experiments, Nanoluc activity was highly comparable across tested conditions (mock and EGFR conditions were virtually identical, while NEF was slightly higher). As a result, normalization to Nanoluc activity would not alter the interpretation of the data nor the significance of the EGFR condition, but would only slightly lower the NEF condition, as such we decided against the normalization.

**Comment:** Stability of nanobody display **Response:** We agree that assessing nanobody stability is of interest. For example, stability could be evaluated by incubating Nb-EVs at 37 °C in serum and monitoring persistence of the nanobody fusion by western blot. While such experiments would provide valuable additional information, they are beyond the scope of the current methodological study.

**Comment:** How does decoration distribution compare between EV proteins when analysed with ONI and nano-flow cytometry? **Response:** We agree that this is an important question, but the used single-EV technology do not provide the resolution required to assess distribution differences at the level of individual proteins.

**Comment:** The authors should demonstrate therapeutic advantages of nanobody decoration.
**Response:** We agree that therapeutic applications are a critical next step. However, the current work was designed as a methodological advance to provide a versatile scaffold for nanobody display and to benchmark against existing strategies. We show that nanobody display enhances EV-cell interactions, which is a prerequisite for future therapeutic applications.

**Reviewer 2**

**Comment 1:** The syndecan approach is not novel; see PMID: 34616047.
**Response:** We thank the reviewer for pointing out this reference. We apologize for not including this reference. We are, of course, fully aware of this paper and, without trying to make excuses for this oversight, we missed adding this reference because, although the syndecan-1 data are shown in a figure, they are never discussed in the main text. We now cite this work and clarify that while syndecan fragments have previously been used to display decoy moieties, our study introduces several novel design elements: the signal peptide of SDC1, the use of the SDC1-CTF fragment with its membrane-spanning domain, and a protease-resistant linker to ensure stable nanobody presentation. This design allows efficient surface display while minimizing proteolytic cleavage, which was not addressed in the cited study.

**Comment 2:** PTGFRN typically achieves higher coating efficiencies (50–60%); the lack of benchmarking weakens claims.
**Response:** We respectfully disagree that our benchmarking with PTGFRN is insufficient. We directly compared SDC1-CTF and PTGFRN constructs under identical experimental conditions, providing a fair side-by-side assessment. While PTGFRN-based approaches can indeed reach higher coating efficiencies in other studies, we observed additional features in our hands that raise questions about its suitability. Specifically, PTGFRN produced prominent high–molecular weight bands in lysates and lEVs and sEVs, which we excluded as a transient transfection artifact and which may correspond to SDS-insoluble aggregates. Such species could raise quality and safety concerns. In contrast, the SDC1-CTF chimera is small, stable, and did not display such signals, making it a robust and versatile alternative. Although SDC1-CTF coating efficiency was in the 6–20% range, its compact design, protease resistance, and homogeneous distribution represent complementary advantages compared to PTGFRN-based methods.

**Comment 3:** Uptake assays show internalization but not functional cargo delivery. Moreover, there are no in vivo data to support targeting or biodistribution, limiting translational relevance.
**Response:** We consider uptake to consist of binding and internalization. We have revised to state this explicitly. Indeed this study does not include in vivo data, which are out of the scope of the current study but should be addressed in the future.

________________________________________________________________________________________

We hope that the revisions and clarifications now position the manuscript appropriately for Journal of extracellular biology. We again thank the reviewers for their constructive feedback, which has helped us strengthen both the manuscript and its interpretation.

Sincerely,
Pascale Zimmermann
On behalf of all authors
